# A Review: Understanding Molecular Mechanisms of Antibody-Dependent Enhancement in Viral Infections

**DOI:** 10.3390/vaccines11071240

**Published:** 2023-07-14

**Authors:** Jyoti Sawant, Ajit Patil, Swarali Kurle

**Affiliations:** HIV Drug Resistance Laboratory, ICMR-National AIDS Research Institute, Pune 411026, India; sawantjyoti11@gmail.com (J.S.); 81.ajit@gmail.com (A.P.)

**Keywords:** Antibody Dependent Enhancement (ADE), complement protein C1q, Fc receptor, mechanism of ADE, severe acute respiratory syndrome coronavirus 2 (SARS-CoV-2)

## Abstract

Antibody Dependent Enhancement (ADE) of an infection has been of interest in the investigation of many viruses. It is associated with the severity of the infection. ADE is mediated by non-neutralizing antibodies, antibodies at sub-neutralizing concentrations, or cross-reactive non-neutralizing antibodies. Treatments like plasma therapy, B cell immunizations, and antibody therapies may trigger ADE. It is seen as an impediment to vaccine development as well. In viruses including the Dengue virus (DENV), severe acute respiratory syndrome (SARS) virus, Middle East respiratory syndrome (MERS) virus, human immunodeficiency virus (HIV), Ebola virus, Zika virus, and influenza virus, the likely mechanisms of ADE are postulated and described. ADE improves the likelihood of productively infecting cells that are expressing the complement receptor or the Fc receptor (FcR) rather than the viral receptors. ADE occurs when the FcR, particularly the Fc gamma receptor, and/or complement system, particularly Complement 1q (C1q), allow the entry of the virus-antibody complex into the cell. Moreover, ADE alters the innate immune pathways to escape from lysis, promoting viral replication inside the cell that produces viral particles. This review discusses the involvement of FcR and the downstream immunomodulatory pathways in ADE, the complement system, and innate antiviral signaling pathways modification in ADE and its impact on facilitating viral replication. Additionally, we have outlined the modes of ADE in the cases of different viruses reported until now.

## 1. Introduction

The antibodies produced against a particular pathogen provide protection against it and facilitate the elimination of that pathogen. On the contrary, they might make it easier for viruses to enter the cells and replicate in the absence of specific viral receptors.

The activity of antibodies produced during disease or following immunization is either neutralizing or non-neutralizing. The neutralizing antibody counters the virus either by obstructing the virus-host cell interaction or by displaying the antigen to the effector cells [1]. The non-neutralizing antibodies are mainly involved in ADCC (Antibody-Dependent Cellular Cytotoxicity), ADCP (Antibody-Dependent Cellular Phagocytosis), and CDC (antibody-medicated Complement-Dependent Cytotoxicity) [2]. Non-neutralizing antibodies are occasionally advantageous to the viruses to ease their entry and replication in the cell. This phenomenon is called ADE, and it was first documented by Hawkes in 1967 [3]. Antibody-dependent enhancement through the FcR was first reported in the case of DENV infection [4]. Non-neutralizing antibodies and various viral serotypes may both contribute to the antibody-dependent aggravation of the disease.

In the case of DENV, ADE is responsible for the disease escalation upon secondary infection with other serotypes [5]. Studies have shown that the disease is exacerbated by the presence of non-neutralizing antibodies that are produced during the main infection or after vaccinations [6]. Other than DENV, Japanese encephalitis virus (JEV), HIV, Ross River virus, West Nile virus (WNV), and Ebola virus, respiratory infections such as respiratory syncytial virus (RSV), SARS-CoV, MERS-CoV, feline infectious peritonitis virus (FIPV), porcine reproductive and respiratory syndrome virus (PRRSV), and measles have also been linked to ADE [7,8,9,10,11,12]. The Mechanism of the ADE is hypothesized and elucidated in the different viruses, but there are still numerous unresolved questions [13]. In the SARS-CoV-2 instance, monoclonal antibody infusion therapy was given to a 58-year-old patient with a history of obesity who was not immunised. This resulted to an ADE, which was then followed by a sharp rise in COVID-19 pneumonia [14].

The antibody disease enhancement takes place through two mechanisms: (i) Extrinsic ADE, where antibodies mediate the increased virus uptake by the cells expressing the FcR, mainly phagocytic cells. The extrinsic mechanism of ADE is responsible for the increased infection through the interactions between the FC region of antibodies bound to the virus and the FcR; and (ii) Intrinsic ADE, in vivo disease enhancement, and excessive immune activation are due to Fc-mediated effector functions. The intrinsic ADE manifests as an inflammatory reaction, a decrease in the innate immune response, and a promotion of viral proliferation. Various ADE mechanisms have been uncovered over the years, with reports of FcR- and complement-mediated ADE involvement [15]. It can be challenging to understand the multifaceted molecular ADE mechanism. The currently hypothesized molecular mechanisms of ADE are the IgG and IgM-mediated entrance and replication of the virus into FcR-expressing cells, antiviral gene suppression, and increased C1q complement activation by the immune complex of virus and antibody [16,17].

FcR-mediated ADE is the most common mechanism of ADE. The IgG antibody-virus complex’s attachment to cells that express FcRs is the primary step in the Fc-mediated ADE. A well-known example of Fc-mediated ADE is DENV; it happens when the virus-antibody complex internalizes through FcRs, followed by viral replication in more types of cells, leading to high viral production due to an increasing number of target cells [18]. Okuya et al. investigated two ADE approaches for SARS-CoV-2, namely ADE mediated by the FcγR and complement component C1q, by using IgG-positive sera for SARS-CoV-2 [19]. FcγRIIb-mediated ADE occurs in instances of SARS-CoV-2, according to a study by Wang et al. [20]. In the case of HIV, the complement receptor makes it easier for antibodies to connect to the virus and/or complement complexes on cells with the FcR or complement receptor, resulting in the fusion of HIV to the target cells, which consequently leads to complement activation and increased production of the virus [21]. In order to carry out the viral entry of MERS-CoV, the antibody/Fc-receptor complex imitates the viral receptor [22]. In ADE, some alterations impact intracellular signaling pathways. The antiviral response gets disabled during the intrinsic ADE, resulting in increased virion production in the infected cells. FcγRIIa interaction with the immune complex activates the immune suppressive response by expressing IL-6, TNF, and IL-10 and by downregulating the expression of Type I interferon (IFN) [23].

## 2. FcγR Signaling Pathways and ADE

The FcR is an antibody receptor expressed by many immune cells, including myeloid and lymphoid cells, particularly macrophages, monocytes, neutrophils, dendritic cells, B cells, and natural killer cells [24]. IgG antibodies attach to the FcγR and neonatal FcRn, while IgA and IgE antibodies bind to FcαR, FcµR and FcεR, respectively. The Roman number in the FcR name denotes the binding affinities for the immunoglobulin; for example, FcRI and IV are high-affinity receptors, and FcRII and III are low-affinity receptors [25]. The FcR and antibody interact with different affinities, and these interactions may be a reversible process. Humans primarily have three types of FcRs: I, II, and III. They are expressed differentially on immune cells, as indicated in Table 1 [26]. The Fc-associated glycan structures and the amino acid sequence of IgG subclasses are contributing factors to the specificity and affinity of the Fc region and its receptors [27].

The Fab region of IgG antibodies interacts with viral epitopes, promoting the neutralization of the virus by blocking the virus from adhering to the host cell. Additionally, the interaction of the virus-antibody complex with FcγR stimulates the effector cell responses. FcγR mediates immunological responses such as immune complex clearance, regulation of antibody formation, antigen processing and presentation, and activation of the adaptive immune response via controlling T-cell proliferation and differentiation.

The type of signaling mainly relies on the molecular motif present in the FcR intracellular domain of FcR. Upon interaction between an antibody and a multivalent antigen, an event such as receptor aggregation on the cell membrane triggers the activation of the FcR signaling. The two FcR-associated known motifs are the immunoreceptor tyrosine-based activation motif (ITAM) and the immunoreceptor tyrosine-based inhibition motif (ITIM). The ITAM motif, associated with the intracellular domains of the FcγRI, FcγRIIa, FcγRIIc, and FcγRIIIa, acts as an activating FcR and is necessary for the activation of cell signaling [28]. Whereas the ITIM associated with FcγRIIb acts as an inhibitory FcR [29]. All FcγR, with the exception of FcγRI, follow the same signaling pathways despite considerable structural variations. FcγRI, has a high affinity for monomeric IgG, while other FcγRs interact with multivalent IgG immune complexes.

**Figure 1:**
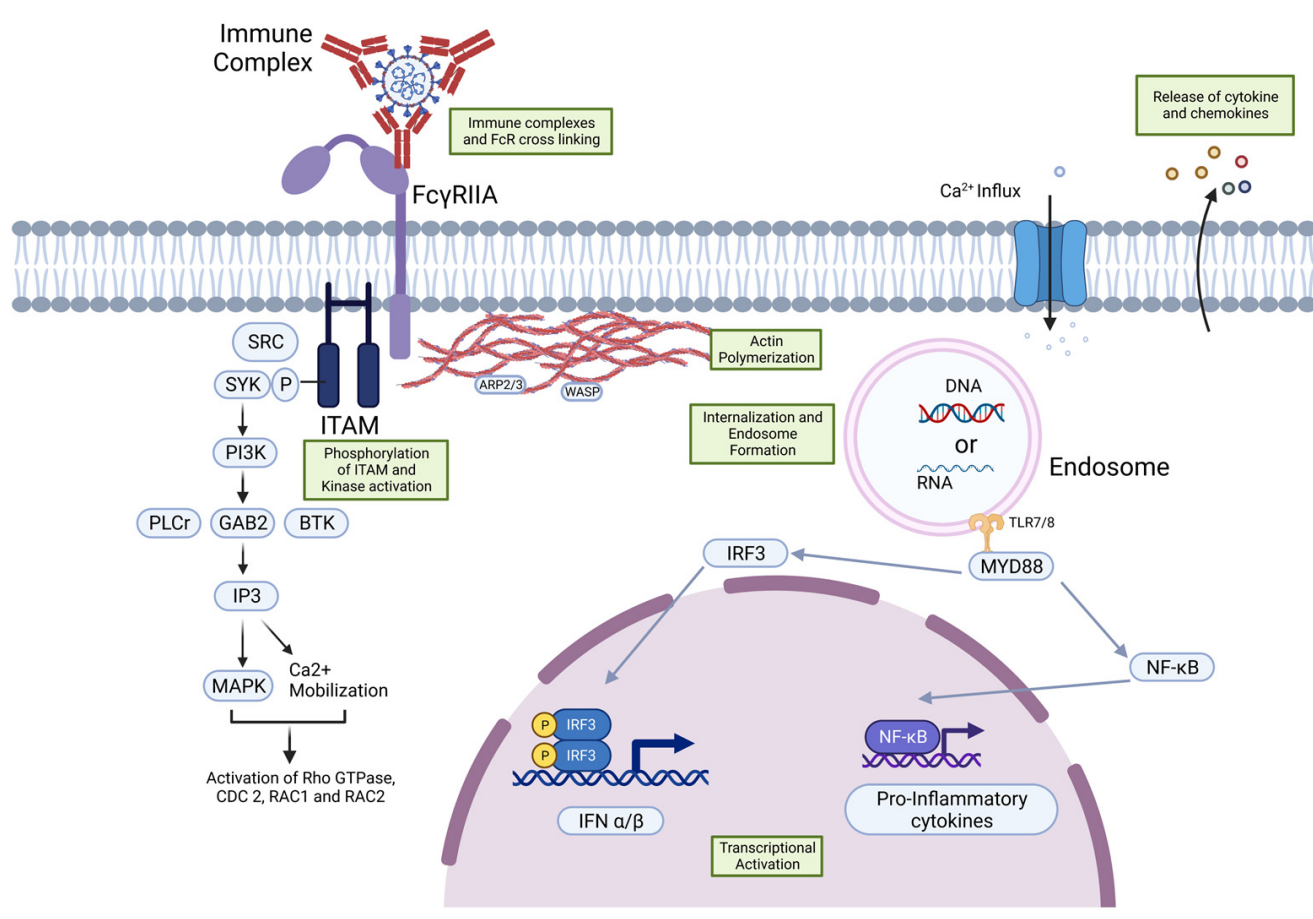
Signaling events occurring after binding of the IgG immune complex to the FcγR immune complex and receptor interactions result in phosphorylation of the immunoreceptor tyrosine activating motifs (ITAMs), followed by activation of the spleen tyrosine kinase (SYK)-mediated PI3K/PKB activation, and SRC family kinases result in the influx of Ca^2+^, which also promotes actin remodeling, which is important for the immune complex phagocytosis and internalization of the receptor. Eventually, the activation of transcription through NF-kB and IRF-3 leads to the expression of pro-inflammatory cytokines.

Aggregation and clustering of FcRs happen during the pathogenic stimulation of IgG, and the ITAM domain is then phosphorylated by the SRC (proto-oncogene tyrosine-protein kinase SRC) or SRC family kinases (SFKs). SRC and SYK kinases (spleen tyrosine kinases) are involved in the immunoreceptor signaling of lymphoid and myeloid cells. The activation of phosphoinositol 3-kinase (PI3K) takes place by the SYK family kinase. Additionally, the activation of later implicated proteins results in the production of inositol triphosphate (IP3). Furthermore, it triggers an influx of Ca^2+^ to activate the protein kinase C (PKC) pathway. Events like the activation of Rho GTPases and the actin polymerization by ARP2/3 and WASP proteins promote receptor internalization and phagocytosis. Finally, the activation of adaptive immunity and the prompt elimination of opsonized virions and infected cells are the outcomes of all these signaling pathways [24]. Figure 1 illustrates these signaling pathway activation events caused by the FcR. To combat ADE, one must be conscious of the mechanisms or pathways that FcR induces.

FcR-mediated ADE utilizes the FcRs phagocytosis pathway to enhance the viral infection. The Dengue virus’s ADE mechanisms have received much attention. According to reports, in the case of DENV, the internalization of the virus-antibody complex takes place via FcγR-expressing cells utilizing the phagocytic FcγR pathway [30]. Moreover, it has been noted that FcγRIIa and FcγRIIIa are active during infection to encourage the DENV ADE. FcγRIIb, nevertheless, was found to be a negative control as it does not trigger intracellular signaling upon aggregation. Studies conducted in vitro utilizing FcγRIIa-expressing BHK cells demonstrated 10-fold greater virus titers than cells lacking the FcγRIIa receptor, affirming the role of FcγRIIa in the ADE of DENV [31]. Human mannose-binding receptor (MR) and DC-SIGN on macrophages serve as DENV’s principal receptors during a typical infection; other proteins like FcR, PI3K, and Rab5 are not implicated; however, they serve as a prerequisite for antibody-mediated DENV entrance [32]. The ADE mechanism of the Ebola virus also involves FcR. A high-affinity association between FcγRs and the antibody Fc portion is observed in the Ebola virus ADE. It is discovered that both the IgG3 and the FcγRIII are heavily engaged in the Ebola ADE [33].

Not only FcγR but also FcαR and FcεR were found to be implicated in the ADE of HIV. Purified serum IgA from HIV patients showed HIV entry in monocytes through FcαR [34,35]. The FcεRI expressing non-permissive cell line HEK 293T showed the internalization and proliferation of PRRSV that were made possible by the ADE mechanism [36].

Non-neutralizing antibodies may contribute to extrinsic ADE when the virus-antibody complex is engulfed by phagocytic cells [37]. Once inside the cell, due to the weak interaction, the virus-antibody complex dissociates from the FcR, which in turn leads to the suppression of the expected immune response. The consequent immunological suppression leads to increased production of cytokines like TNF, IL-6, and IL-10. These cytokines, especially IL-10, suppress the cytokine-expressing genes, which results in the inhibition of type 1 IFN expression, providing the optimal environment for virus replication. Immunological suppression may also lead to the misregulation of autophagy-related proteins (ATG5 and ATG12) and dihydroxyacetone kinases. The signal cascade is influenced by the activation of the negative regulators, which also downregulates the expression of type 1 IFN. As a result, the interferon-related antiviral responses start to malfunction, which eventually makes it more straightforward for the virus to replicate inside the cells [38].

### FcR-Mediated ADE in SARS-CoV-2

The COVID-19 pandemic allowed for expedited vaccine development. Multiple vaccine candidates have been developed and approved for use in situations of emergency. Nevertheless, studies focusing on aspects of vaccine-induced antibody longevity as well as their effect on the manifestation of secondary infections are also necessary [24]. Studies have demonstrated that in the case of coronaviruses like SARS-CoV and MERS-CoV, the ADE phenomenon is mediated through anti-spike IgG antibodies and the FcR [22]. SARS-CoV-2 enters through the SARS-CoV receptor ACE-2 and transmembrane protease serine 2 (TMPRSS2) [39]. Enhanced virus internalization in macrophages, monocytes, and B cells was detected by an ACE-2-independent, IgG/FcγRII dependent mechanism [40,41]. Therefore, it is important to better understand how the FcR plays a role in SARS-CoV-2 ADE. The investigations showed that ADE of SARS-CoV-2 exhibited the involvement of FcγR such as FcγRIIb [20], FcγRIIIa (CD16A), and FcγRIIa (CD32A) [42]. Okuya et al. observed that the FcγR and complement component C1q are accountable for the ADE process. They also noticed that 50% of IgG antibodies that were positive for SARS-CoV-2 and could neutralize the virus showed ADE in the presence of FcγR and C1q [19]. Maemura et al. concluded that the antibodies generated during the COVID-19 convalescent phase were able to ease the ADE through the FcγRIIa and FcγRIIIa. Even so, proinflammatory cytokine or chemokine production in the macrophages was not upregulated [42]. In this respect, the antibodies were implicated in the extrinsic ADE mechanism and not in the intrinsic ADE mechanism. This may imply that there are additional mechanisms besides the FcR-mediated that contribute to the SARS-CoV-2 ADE. According to a study by Wang et al., SARS-CoV-2 ADE occurs via FcγRIIb, an inhibitory FcR. They additionally demonstrated that the ADE mechanism necessitates processes like virus-antibody bivalent interactions, micropinocytosis, and endocytosis for the internalization of the virus [20]. The intrinsic ADE can be evinced from a case study where the cytokine release syndrome (CRS) arises after infusion of an anti-SARS-CoV-2 antibody in a 75 year old patient. An increased level of the IL-6, TNFα, IL-8, and IL-10 cytokines in serum contributed to the immune activation and, finally, the progressive acute respiratory distress syndrome [43]. As a result of the findings from the aforementioned studies, it is important to evaluate the mechanism of ADE in the case of SARS-CoV-2 and its effects on the severity of the disease.

## 3. Complement and ADE

Complement receptors are present on different types of immune cells, including monocytes, macrophages, neutrophils, B cells, follicular dendritic cells, fibroblasts, and smooth muscle cells. In comparison to the FcR, complement receptors are numerous and expressed in a wide range of cell types [44]. The 50 proteins that make up the three complement pathways—the classical, lectin, and alternative pathways—intersect at complement factor C3. The complement system eventually activates the adaptive immune system by inducing inflammation, opsonization, and ultimately lysis of the infected cell. Complement activation occurs through antibody-mediated effector functions, where the complement 1q (C1q) first binds to the virus-antibody complex, followed by the development of C3 to C9 complements and convertases, as well as the membrane attack complex (MAC), which destroys the infected cell.

Target cells undergo a higher cytopathic effect because of complement-mediated ADE, which can negate the protective effects of neutralizing antibodies. According to studies on viruses that are not macrophage-tropic, the formation and deposition of the antigen-antibody-complement complex might increase immunological activation, which in turn increases the release of pro-inflammatory cytokines and worsens illness. Various viruses, notably HIV, Ebola, West Nile, DENV, RSV, and others, have been connected to this complement-mediated ADE [15]. Findings from studies showing elevated levels of C3 cleavage in individuals with Dengue hemorrhagic fever provide convincing proof that complement proteins play a role in ADE [45]. The complement system can facilitate the virus infection of the target cell in two ways: first, by making it easier for the virus to bind to the complement receptor, and second, by promoting the fusion of the viral capsule and cell membrane.

### 3.1. Virus Binding to the Complement Receptor

**Figure 2:**
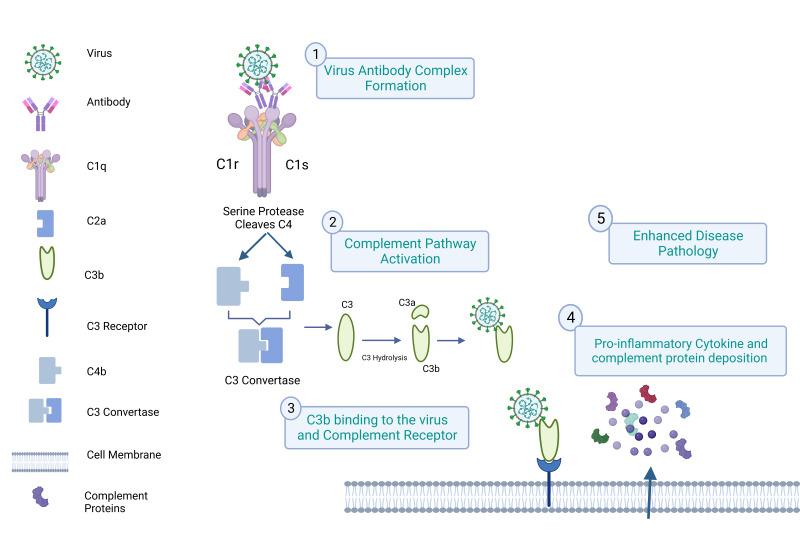
ADE in complement receptor-expressing cells: the virus-antibody complex activates the complement system first by interaction with C1q, followed by activation of C2a and C4b, and finally the production of the C3 convertase, which is the conversion point of all three complement pathways, viz., classical, lectin, and alternative complement activation pathways. The hydrolysis of the C3 produces C3b, interacts with the virus, and complements the receptor on the cell. Finally, the figure shows the lysis of the cell and enhanced disease pathology.

Complement activation essentially governs the effector actions of antibodies. C1q is the first complement that binds to the viral antibody complex. In the process of C1q-mediated ADE, the C1q molecule with serine protease proenzyme, C1r, and C1s are involved in the virus binding to the complement receptors. After the binding of C1q to the virus-antibody complex, C1r and C1s dissociate from C1q. Dissociated C1s cleave the C2 and C4, and the formation of the C3 convertase cleaves the C3, leading to the activation of the C3 and its receptor present in the cell [46]. Figure 2 shows a schematic representation of all the events, including ADE in complement receptor-expressing cells.

A complement-mediated ADE mechanism has been reported in West Nile and HIV. In this complement-mediated ADE phenomenon, C1q binds to the glycoprotein 41 (gp41) present on the membrane of HIV. Monocytes, macrophages, B cells, neutrophils, and other cell types express C1q receptors. So at the early stage of infection, the sub-neutralizing concentration of the antibodies increased monocyte infection in humans [47]. Additionally, it was observed that, in the presence of C1q, ADE antibodies boosted viral attachment; this example shows the levels of C1q are an important factor in blocking ADE.

### 3.2. Virus Capsule and Cell Membrane Fusion Mechanism

Another ADE C1q-dependent mechanism involves the interaction of virus-antibody complexes with C1q, which results in the viral capsule and cell membrane fusion occurring through the interconnection of C1q and its receptor [48]. The binding of the antibody-virus-C1q complex to the C1q receptor triggers the intracellular signaling pathways, assists the binding of the virus and its receptor, and finally ends in endocytosis.

In contrast to the complement system’s defense strategy, the Ebola virus causes C1q to mediate ADE in human kidney cells by internalizing the virus-antibody-C1q complex by cell membrane fusion. It increases the possibility of the attachment of the viruses to C1q receptors and their entry inside the cell [17]. It was also discovered that the virus-antibody and C1q complex interactions with the C1q receptor activate pathways including Wnt/-catenin, PI3K, and certain tyrosine kinases [49]. The potential contribution of these pathways to ADE caused by C1q must thus be addressed in more detail.

#### C1q-Mediated ADE in SARS-CoV-2

A study by Okuya et al. showed that C1q- and/or FcγR-mediated ADE activities could be observed in the serums collected from COVID-19 convalescent patients as well as acute phase patients [19]. Given that the respiratory epithelial cells that are mostly infected with SARS-CoV-2 have the C1q receptor and that a higher plasma concentration of C1q may have beneficial effects, there must be a greater clinical impact of C1q-mediated ADE. In the case of severe COVID-19 patients, the relationship between disease severity and an increased level of C3a was noticed [50]. Additionally, the excessive activation of the complement system is what causes elevated levels of complement products like C3a and C5a.

Targeting the precise pathways to prevent disease enhancement might benefit from better knowledge of the role of complement in mediating ADE. The development of various vaccine strategies and antibody treatments to successfully manage subsequent illness enhancement might benefit from research on mechanisms to modify the pathways.

## 4. Antiviral Activity and ADE

Studies on molecular signaling clarify our understanding of the mechanisms behind the ADE phenomenon. In response to viral infection, two pathways of innate immunity are activated: a toll-like receptor (TLR)-dependent and a TLR-independent pathway. The infection of RNA viruses activates signaling pathways leading to the secretion of type I IFN and finally the activation of transcription factors like nuclear factor kappa-light-chain-enhancer of activated B cells (NF-κB), interferon-regulatory factor 3 (IRF3), and interferon-regulatory factor 7 (IRF7) [51]. In TLR-independent signaling, the retinoic-acid inducible gene I (RIG-I) and the melanoma differentiation-associated gene 5 (MDA5) recognize the viral RNA in the cytoplasm, which activates the mitochondrial antiviral signaling protein (MAVS), the mitochondrial signaling adaptor. Finally, type I IFN signaling pathways are activated when other kinases, such as TANK-binding kinase 1 (TBK1) and IKKi, bind to MAVS and phosphorylate IRF3/IRF7 [52].

### 4.1. Antiviral Response upon Virus Entry through Its Receptor

The prevention of viral replication is brought on by antiviral action, which is associated with type I IFNs. To comprehend the antiviral response within the cell, we can take into consideration a well-researched example, as antiviral activity arises upon the entry of DENV through the receptor. The first virus is detected by the toll-like receptors TRL-3 and TRL-7 inside the cell; if viral RNA escapes the endosome, MDA5 and RIG-I detect it and trigger a TLR-independent pathway. Which leads to the expression of pro-inflammatory cytokines and the activation of signal transducer and activator of transcription 1 (STAT 1). Nitric oxide radicals are produced as a final byproduct of pathway activation, which in turn inhibits DENV replication [53,54].

### 4.2. Antiviral Response and ADE

**Figure 3:**
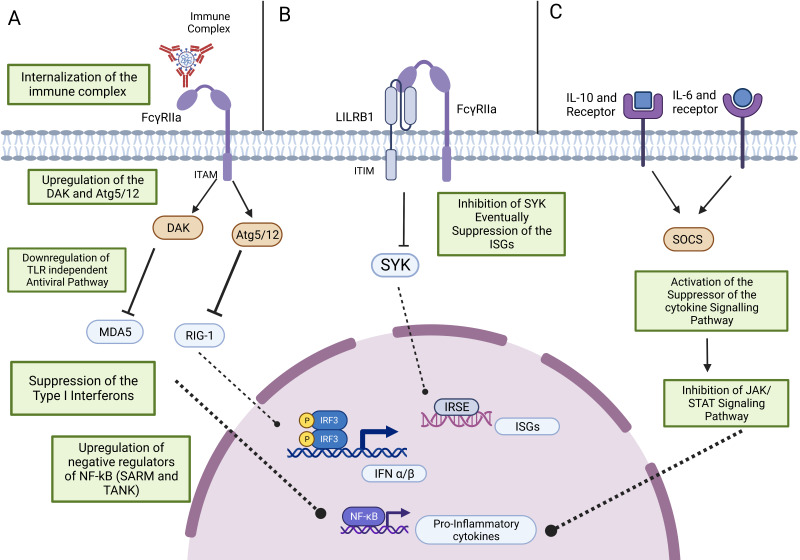
Antiviral response in the ADE Mechanism. (**A**) Upregulation of the DAK and Atg5/12 and downregulation of the MDA5 and RIG-1, which are the components of the TLR-independent antiviral pathway, results in the suppression of type-1 interferon and proinflammatory cytokine production. (**B**) An immune complex with FcγRIIa co-ligate to LILRB1 inhibits SYK activation, and revocation of the ISGs expression takes place. (**C**) IL-10 produced after FcR and immune complex interaction triggers the SOCS and inhibits pro-inflammatory cytokine production by impeding the JAK/STAT signaling pathway.

Antiviral responses, which suppress immune activation, contribute to intrinsic ADE. The potential three ways that are responsible for suppressing the antiviral immune response can be listed as follows: first, downregulation of genes RIG-I and MDA5, which are components of the TLR-independent antiviral pathway, and upregulation of dihydroxyacetone kinase (DAK) and autophagy-related 5–autophagy-related 12 (Atg5/12) [38]. Because of this altered gene expression pattern, the suppression of the expression of type I IFNs was noted (Figure 3A). Second by modifying the expression of the cytokines. Upregulation of TNF, IL-6, and IL-10 has been observed in the monocytes, macrophages, and DCs that were infected. The elevated level of IL-10 activates the suppressor of the cytokine signaling pathway and eventually the JAK/STAT pathway (Figure 3C). The nitric oxide synthase gene was found to be downregulated due to the increased production of IL-10 [54]. Third by inactivating the SyK and suppressing the interferon-stimulating genes after co-ligation of the FcγR and leucocyte immunoglobulin-like receptor B1 (LILRB1) (Figure 3B) [23].

During ADE by DENV, the secretion of proinflammatory cytokines and type I interferon production are targeted to suppress the immune system. Genes such as IRF-1, NOS-2, RIG-I, and MDA-5 are downregulated during the ADE, which is contradictory to normal DENV infection [55]. Downregulation of the type I interferon-stimulated gene (ISG) induces the co-ligation of LILRB1, a tyrosine-based inhibition motif-bearing immunoreceptor, which leads to dephosphorylation of the spleen tyrosine kinase, resulting in the downregulation of the type I ISG [56].

During secondary infection, or ADE, by DENV, the suppression of TLR expression and signaling has been reported by Modhiran et al. This suppression was mediated by the TAF family-associated NF-κB activator (TANK) and sterile-alpha and armadillo motif-containing proteins (SARM), which are negative regulators of TLR. Additionally, during the ADE in DENV, TLR-3, 4, and 7 were found to be downregulated [57]. It has also been concluded that during ADE, differentially expressed genes (DEGs) are upregulated. Mainly the genes that are involved in vesicular transport and mRNA processing. Contrastingly, receptor-mediated viral entry into the cells shows the downregulation of the DEGs-like genes responsible for host protein translation [37]. A study by Hueston et al. demonstrated the downregulation of IFNβ and nitrogen intermediates in macrophages during Zika virus infection [58]. Additionally, it is also observed that increased endocytosis suppresses the expression of the antiviral genes. This phenomenon of ADE is observed in the Ross River virus, where the viral entry is mainly through the FcR instead of the viral receptor. The replication of the virus affects the TNFα, nitric oxide synthase 2 (NOS2), and IFN regulatory factor 1 (IRF-1) antiviral genes and promotes immune escape of the virus. The replication of the virus influences the antiviral transcription factors [59]. In summary, these investigations have indicated that the primary immune suppression targets are type I IFN and the generation of pro-inflammatory cytokines.

## 5. ADE Assays

In vitro ADE assays are the measures of diligent assessment of the vaccines and the targeted drugs. In vitro ADE studies are carried out in the presence of viruses or pseudoviruses and antibodies, and human FcR-expressing cell lines such as K562, Mylc, Raji, Vero E6, and BHK-21 are utilized. Techniques frequently utilized for detecting the ADE effect on virus infection in FcR-expressing cells include flow cytometry, plaque assays, qPCR, and luminometry. The ADE assays performed using cell lines, virus isolates, or pseudo-typed viruses are listed in Table 2.

In order to build a rapid ADE assay system for dengue virus infection, Yamanaka et al. used recently developed chimeric viral particles called single-round infection particles (SRIPs) [60].

In vitro ADE assays cannot reflect the exact in vivo immune status, but they can provide beneficial information about the risk assessment. The results obtained are even contradictory in both conditions. Dapeng Li et al. conducted a study by using in vitro infection-enhancing and neutralizing antibodies in mouse and macaque models. Out of 46 monkeys, one showed alveolar and perivascular edema, and three showed enhancement of lung pathology [61]. The results drawn from the in vitro ADE experiments, which use a single kind of immune cell, do not anticipate the heightened infection in vivo since the in vivo environment is complex and, at the same time, various mechanisms are involved in managing and eliminating the disease. Additionally, while using the model system, we must consider that the FcR expression system is different from humans.

**Table 2:** ADE assays performed using the different cell lines, viruses, or pseudo-typed viruses.

Virus Studied	Cell Line	Pseudovirus	Reporter System	Readout	References
DENV	K562 and Mylc cells.	Single-round infection particles (SRIPs).	Luciferase.	Luminometer.	[60]
Ebola	Human embryonated kidney (293) cells (HEK293) or Vero E6	Pseudotyped vesicular stomatitis virus (VSV) and VSVΔG-EBOV GP.	GFP.	Fluorescent microscopy.	[11]
HIV	CD21-expressing cell line and T cell line naturally expressing complement receptor 2 (CR2; CD21).	HIV isolates and simian-human immunodeficiency viruses (SHIVs).	Intracellular staining for P24 expression.	Flow cytometry.	[62]
SARS-CoV-2	Raji cells, K562 cells, primary B cells, and Vero E6.	Spike protein expressing pseudovirus and VSV pseudotyped with SARS CoV-2 S (VSV-SARS2).	Luciferase and GFP.	Luminometer, flow cytometry, and fluorescent microscopy.	[20]

## 6. SARS-CoV-2 Vaccines and ADE Mechanism

The ability of the vaccines to elicit an immune response to the antigen will make them effective in repeated encounters. As we have already mentioned, the mechanism of ADE makes the development of vaccines very challenging. Currently, immunodominant epitopes are exploited to overcome ADE in SARS-CoV-2 that correspond to spike protein since it is involved in virus entry and is the primary target of antibodies. The N-terminal domain (NTD) and the receptor-binding domain (RBD), two highly immunogenic domains of the S1 subunit of spike protein, are the targets of both polyclonal and monoclonal antibodies [63,64]. Additionally, because virus-encoded RNA-dependent RNA polymerases lack proofreading ability, RNA viruses are vulnerable to mutations. The SARS-CoV-2 spike protein mutations significantly increase infectivity [65].

Omicron is currently the most prevalent circulation variant and carries 30 mutations. The Omicron subvariants were shown to be much less recognized and neutralized by antibodies from 46 vaccine recipients [66]. Therefore, we must take into consideration how virus evolution may impact the efficacy of spike protein-directed vaccines as well as whether it may provide an escape strategy for newly altered viruses through ADE [67]. Likewise, a study by Shimizu J. et al. examined serum samples from vaccinated individuals and found ADE activity as well as a failure to display neutralization against the Omicron strain [68].

Further, antibodies against the variable region of the RBD domain promoted the SARS-CoV-2 infection revealed through peptide array scanning analyses. Fan Wu et al. revealed that antibodies lacking ADE bind to the spike protein with three “up” RBDs, while antibodies promoting ADE bind to the spike protein in a shift-angled manner with one “up” and two “down” RBDs [69]. To design a vaccine that is effective against the highly mutating SARS-CoV-2, it is vital to monitor mutations while taking all these factors into consideration.

The use of the entire SARS-CoV-2 virus as an immunogen in vaccines is another topic to be explored in the context of ADE. These inactivated vaccines will predominantly result in non-neutralizing antibodies against the non-spike protein, which is extremely conserved. Consequently, it can be a concern for possible ADE upon future infection with the novel variants [70]. By using the mRNA, DNA, and protein component vaccines, polysaccharide-RBD-conjugated nanoparticle vaccine [71], or beta-glucan [72], ADE can be avoided by preventing the production of non-neutralizing antibodies or antibodies against the other structural proteins after immunization.

## 7. Conclusions

The current pandemic of SARS-CoV-2 has led to different studies on the mechanisms of ADE and strategies to control the spread. Assessing the extent of protection provided by antibodies produced after vaccination or in response to a natural infection was an important component of these studies. However, evidence from other viruses in terms of ADE has shown a destructive role of antibodies, and studies on SARS-CoV-2 are currently limited. ADE is an important aspect as it is a reason for the failure of vaccines as well as antibody therapies; hence, it needs in-depth and absolute knowledge. Different mechanisms contributing to ADE in the case of SARS-CoV-2 need to be studied comprehensively. In addition, there is a demand to design strategies to control ADE and vaccine-associated enhanced respiratory diseases (VAERD).

To overcome ADE during clonal antibody therapies, approaches like controlled dose, changing of antibody target, and usage of some inhibitors can be considered. The modification of the antibodies, like the LALA and YTE mutations and TM modification in the anti-SARS-CoV-2 monoclonal antibody FC region, prevented the antibody-dependent enhancement by decreasing the binding affinity to FcγR and C1q [73,74]. Additionally, highly fucosylated antibodies were found to be helpful in overcoming ADE through FcRs [75]. To prevent ADE after vaccination, Sun et al. developed a polysaccharide-RBD-conjugated nanoparticle vaccine. In mouse models, this vaccine has induced potent efficacy against SARS-CoV-2. Additionally, after stimulating antibodies with a vaccine in rhesus macaques, neutralizing antibody titers were reported to show no signs of antibody-dependent enhancement [71]. In the treatment of monoclonal antibodies, the modulation of Fc glycosylation has been demonstrated to be a useful method for preventing ADE since it preserves the effector function, which is lost in Fc mutation methods. These discoveries may have an influence on ADE-prone viruses like SARS-CoV-2 [76].

The cytokine storm in COVID-19 was caused by cross-reactive antibodies that could result in ADE, and inhibitors like rapamycin (an mTOR inhibitor) can be recommended as treatments due to their negative effects on memory B cell activation [77]. In addition, by preventing the translation of structural proteins and the SARS-CoV2 viral polymerase, it prevents the growth and replication of the virus and the autophagy of the infected cells [78]. ADE depends on the neutralization capacity and concentration of the antibody; hence, the immunization regimen needs to assess antibody responses to ADE.

In conclusion, an in-depth understanding of the ADE process enables oversight of the development of novel drugs, antibody treatments, and vaccines. In this review, the ADE processes are outlined by incorporating aspects like FcR, or complement receptor-mediated viral entry, altered pathways, and antiviral response. Our review offers insights into the examination of the potential risks connected with ADE, particularly in the context of the COVID-19 pandemic or other emerging diseases.

## Figures and Tables

**Table 1:** Expression pattern of FcγR in different immune cells, its function, and its participation in the ADE of different viruses.

	FcγRI	FcγRIIa	FcγRIIb	FcγRIIc	FcγRllla	FcRIIIb
	(CD64)	(CD32a)	(CD32b)	(CD32c)	(CD16a)	(CD16b)
Expressed by human immune cells	Macrophages, Eosinophils, Neutrophils, and Dendritic cells.	Platelets, Macrophages, Neutrophils, and Eosinophils.	Platelets, Dendritic cells, B cells, Mast cells, Neutrophils, and Eosinophils.	Natural Killer cells depend on allele status.	Natural Killer cells, Macrophages, Neutrophils, and Eosinophils.	Macrophages, Neutrophils, and Follicular dendritic cells.
Binding specificity	IgG1, IgG3, and IgG4.	IgG.	IgG.	IgG.	IgG1 and IgG3.	IgG1 and IgG3.
Functions	Phagocytosis, respiratory burst activation, and cell activation.	Degranulation, Phagocytosis, and ROI production.	Inhibits the phagocytosis and release of the pro-inflammatory cytokines, Degranulation, platelet activation, and B cell activation.	Phagocytosis and clearing of immune complexes.	Antibody-dependent cell-mediated cytotoxicity (ADCC) initiation, and cytokine release.	Phagocytosis, cytokine, and chemokine release.
ADE participation of viruses	DENV, Ebola virus, PRRSV, and JEV.	DENV, FIPV, MERS-CoV, SARS-CoV, and WNV SARS-CoV-2.	SARS-CoV-2, DENV, WNV, and PRRSV.	Unknown.	DENV, Ebola virus, HIV, PRRSV, and JEV.	Unknown.

## Data Availability

No new data were created or analyzed in this study. Data sharing is not applicable to this article.
